# Antioxidant activity and mechanism of Rhizoma *Cimicifugae*

**DOI:** 10.1186/1752-153X-6-140

**Published:** 2012-11-23

**Authors:** Xican Li, Jing Lin, Yaoxiang Gao, Weijuan Han, Dongfeng Chen

**Affiliations:** 1 School of Chinese Herbal Medicine, Guangzhou Higher Education Mega Center, Guangzhou University of Chinese Medicine, Waihuang East Road No.232, Guangzhou 510006, China; 2 School of Basic Medical Science, Guangzhou University of Chinese Medicine, Guangzhou 510006, China

**Keywords:** Rhizoma *Cimicifugae*, 升麻, Antioxidant activity, Metal chelating, Radical-scavenging, Phenolic acid

## Abstract

**Background:**

As a typical Chinese herbal medicine, rhizoma *Cimicifugae* (RC, 升麻 in Chinese) possesses various pharmacological effects involved in antioxidant activity. However, its antioxidant activity has not been reported so far. The aim of the present study was to systematically evaluate the antioxidant ability of RC *in vitro,* then discuss the mechanism.

**Methods:**

Firstly, five RC extracts (i.e. petroleum ether extract PERC, ethyl acetate extract EARC, absolute ethanol extract AERC, 95% ethanol extract 95ERC, and water extract WRC) were prepared and determined by various antioxidant methods, including anti-lipidperoxidation, protection against DNA damage, ·OH scavenging, ·O_2_^-^ scavenging, DPPH· (1,1-diphenyl-2-picryl-hydrazl radical) scavenging, ABTS^+^· (2,2’-azino-bis (3-ethylbenzo- thiazoline-6-sulfonic acid radical ion) scavenging, Cu^2+^-chelating, and Fe^3+^ reducing assays. Subsequently, we measured the chemical contents of five RC extracts, including total phenolics, total saponins, total sugars, caffeic acid, ferulic acid and isoferulic acid. Finally, we quantitatively analyzed the correlations between antioxidant levels (1/IC_50_ values) and chemical contents.

**Results:**

In the study, the antioxidant levels and chemical contents (including total phenolics, total saponins, total sugars, caffeic acid, ferulic acid and isoferulic acid) of five RC extracts were determined by various methods. In all antioxidant assays, five RC extracts increased the antioxidant levels in a dose-dependent manner. However, their antioxidant levels (IC_50_ values) and chemical contents significantly differed from each other. Quantitative analysis of the correlation showed that total phenolic was of significant positive correlations (average *R* value was 0.56) with antioxidant levels; In contrast, total sugars and total saponins had no positive correlation with antioxidant (the average R values were −0.20 and −0.26, for total sugars and total saponins, respectively); Among total phenolics, three phenolic acids (caffeic acid, ferulic acid and isoferulic acid) also displayed positive correlations (the average R values were 0.51, 0.50, and 0.51, for caffeic acid, ferulic acid and isoferulic acid, respectively).

**Conclusions:**

As an effective antioxidant, Rhizoma *Cimicifugae* can protect DNA and lipids against oxidative damage. Its antioxidant ability can be responsible for its various pharmacological effects and may be mainly attributed to the existence of total phenolics, among which caffeic acid, ferulic acid and isoferulic acid are regarded as main bioactive components. Rhizoma *Cimicifugae* exerts its antioxidant effect through metal-chelating, and radical-scavenging which is via donating hydrogen atom (H·) and donating electron (e).

## Background

As we know, reactive oxygen species (ROS) are various forms of activated oxygen including free radicals and non-free-radical species. ROS can oxidatively damage vital cellular structures such as lipids and DNA [1,2], then lead to severe biological consequences including mutation, cell death, carcinogenesis, and aging [3].

Therefore, it is important to search for potential therapeutic agents for oxidative damage. In recent years, medicinal plants especially Chinese medicinal herbals have attracted much attention.

As a typical Chinese herbal medicine, rhizoma *Cimicifugae* (RC, 升麻 in Chinese, the photo is shown in Additional file 1) has been used for over 2000 years [4]. From the viewpoint of tradition Chinese medicine (TCM),RC can elevate *yang*, lift *qi*, clear *heat,* remove *toxic,* induce sweats to dispel exopathogens, and promote eruption [5].

Modern medicine indicated that RC possessed various pharmacological effects. Ye reported that RC possessed antidepressant-like properties in rodents [6]; Kim pointed out that RC can treat pain and inflammation [7]; An isopropanolic extract of RC, however, was proved to be able to diminish the urinary content of PYR and DPY and the morphometric correlates of bone loss associated with ovariectomy in rats [8]; The supply of RC can therefore prevent OVX-induced bone loss in mice [9]. In addition, the extract of RC was found to have protective effect against gastric injury [10]. According to free radical biology & medicine [11], these pharmacological effects are related to antioxidant ability. However, its antioxidant ability has not been explored so far.

Therefore, the aim of the study was to investigate the antioxidant ability of RC *in vitro*, then further discuss the antioxidant mechanism.

## Results and discussion

As an important biomolecule, lipid can be easily attacked by ROS to generate lipid peroxidation which is harmful to cell. For example, the highly reactive ·OH radical can attack to lipid to produce lipidperoxidation (Equations 1–3):

(1) $$\;\mathrm{Lipid}-H+\cdot \mathrm{OH}\to \mathrm{Lipid}\cdot +H_{2}O$$

(2) $$\;\mathrm{Lipid}\cdot +O_{2}\to \mathrm{Lipid}-O_{2}\cdot$$

(3) $$\;\mathrm{Lipid}-H+\mathrm{Lipid}-O_{2}^{\cdot }\to \mathrm{Lipid}-O_{2}H+\mathrm{Lipid}\cdot$$

In our study, five RC extracts increased the anti-lipidperoxidation percentages in a dose-dependent manner (Additional file 2). It means that five RC extracts can effectively protect lipid against oxidative damage. Among them, EARC possessed the highest anti-lipidperoxidation activity (Table 1).

**Table 1:** **The IC**_**50**_**values of five RC extracts and the positive controls (μg/mL)**

**Assays**	**PERC**	**EARC**	**AERC**	**95ERC**	**WRC**	**GSH**
Anti-lipid peroxidation	13.99 ± 2.11^e^	4.55 ± 0.44 ^b^	14.92 ± 4.31 ^d^	10.62 ± 0.97 ^c^	19.18 ± 2.04 ^f^	0.028 ± 0.00 ^a^ *
DNA protective effect	-	1905.51 ± 177.54 ^c^	14397.18 ± 1075.01 ^d^	1090.11 ± 21.25 ^b^	-	114.42 ± 2.66 ^a^ **
·OH	561.19 ± 21.16 ^f^	111.55 ± 1.32 ^c^	132.32 ± 0.15 ^d^	84.30 ± 1.02 ^b^	233.45 ± 15.70 ^e^	37.67 ± 0.67 ^a^ **
·O_2_^-^	322.81 ± 14.90 ^b^	306.68 ± 0.87 ^b^	284.36 ± 11.43 ^b^	301.08 ± 8.34 ^b^	650.65 ± 59.55 ^c^	81.65 ± 5.21 ^a^
Chelating	191.86 ± 1.84 ^b^	319.20 ± 6.36 ^c^	872.71 ± 30.63 ^e^	553.34 ± 12.59 ^d^	2289.11 ± 100.00 ^f^	107.86 ± 0.31 ^a^ ***
DPPH·	813.00 ± 225.17 ^d^	227.09 ± 10.14 ^b^	260.34 ± 11.37 ^c^	254.73 ± 4.33 ^c^	229.17 ± 5.81 ^b^	5.13 ± 1.19 ^a^ **
ABTS·^+^	395.65 ± 77.34 ^e^	72.10 ± 1.32 ^b^	96.39 ± 1.12 ^d^	94.38 ± 2.45 ^d^	83.58 ± 0.81 ^c^	4.76 ± 0.28 ^a^
Fe^3+^ reducing	533.12 ± 54.25 ^e^	159.35 ± 1.37 ^b^	317.06 ± 3.47 ^c^	156.19 ± 2.12 ^b^	488.75 ± 18.6 0 ^d^	51.89 ± 1.29 ^a^

IC_50_ value is defined as the concentration of 50% effect percentage and calculated by linear regression analysis and expressed as mean ± SD (*n* = 3). The linear regression was analyzed by Origin 6.0 professional software. Means values with different superscripts in the same row are significantly different (*p <* 0.05), while with same superscripts are not significantly different (*p <* 0.05)**.** *The positive control was BHA, instead of GSH. ** The positive control was Trolox, instead of GSH. *** The positive control was Sodium citrate. Results were analyzed by independent samples. *PERC*, petroleum ether extract of rhizoma *Cimicifugae; EARC*, ethyl acetate extract of rhizoma *Cimicifugae; AERC*, absolute ethanol extract of rhizoma *Cimicifugae; 95ERC*, 95% ethanol extract of rhizoma *Cimicifugae; WRC*, water extract of rhizoma *Cimicifugae.* --: Cannot be detected.

**Scheme 1:**
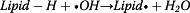
The reaction of MDA (malondialdehyde) and 2-thiobarbituric acid (TBA).

Besides lipid, another biomolecule DNA can also be oxidatively damaged by ROS (especially ·OH). It is well known that DNA consists of deoxyribose, organic phosphate and various base pairs. When DNA is attacked by ·OH radical, MDA (malondialdehyde) and a number of oxidative lesions are generated [12]. MDA combines with 2-thiobarbituric acid (TBA) to produce thiobarbituric acid-reactive substances (TBARS) with λ_max_ at 530 nm (Scheme 1).

Therefore, the A_532nm_ value is proportional to the produced amount of ·OH radicals. Higher A_532nm_ values indicate higher levels of ·OH radicals. If an antioxidant sample is added, the A_532nm_ value will decrease, suggesting that some ·OH radicals are scavenged and the hydroxyl-induced DNA damage are protected by the antioxidant.

Among five RC extracts, 95ERC and EARC were proved to be of protective effect against hydroxyl-induced DNA damage (Table 1 and Additional file 2).

Previous studies have shown that there are two approaches for natural phenolic antioxidant to protect DNA oxidative damage: one is to scavenge the ·OH radicals then to reduce its attack; one is to fast repair the deoxynucleotide radical cations resulting from ·OH radicals attack [11]. In order to further confirm whether the protective effect against DNA oxidative damage was relevant to radical-scavenging ability, the ·OH and ·O_2_^-^ radical-scavenging abilities of five RC extracts was determined *in vitro.*

As we know, ·OH radical in body is produced by Fenton reaction (Equation 4). However, our data suggested that five RC extracts can effectively scavenge ·OH radicals (Table 1 and Additional file 2).

(4) $$\;Fe^{2+}+H_{2}O_{2}\to \cdot \mathrm{OH}+OH^{-}+Fe^{3+}$$

Besides ·OH radical, superoxide anion radical (·O_2_^-^) is also regarded as one important form of ROS in living cell. It can directly attack DNA or lipid [13], and can transferred into ·OH via Haber-Weiss reaction (Equation 5) to indirectly damage DNA or lipid as well [2]. The results in Table 1 and Additional file 2 showed that five RC extracts could also scavenge ·O_2_^-^ radicals.

(5) $$\;\cdot O_{2}^{-}+H_{2}O_{2}\overset{\mathrm{Iron}\;\mathrm{ion}}{\to }\cdot \mathrm{OH}+OH^{-}+O_{2}$$

Taken together, the protective effect of RC against hydroxyl-induced DNA oxidative damage can be assumed to be relevant to the ·OH or ·O_2_^-^ radical-scavenging ability.

**Scheme 2:**
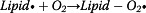
The proposal reaction of phenolic acids binding Fe^2+^.

However, as illustrated in Equations 4 and 5, the generations of ·OH and ·O_2_^-^ radicals rely usually on the catalysis of transition metals (especially Fe and Cu). So we further explored the metal chelating abilities of five RC extracts. The dose–response curves showed that five RC extracts possessed effective metal chelating abilities (Additional file 2). Our previous reports have shown that the chelating ability might be mainly attributed to *ortho* dihydroxyl groups in phenolic compounds [14]. For example, phenolic acids can bind Fe^2+^ as the following mechanism (Scheme 2) [15].

The fact that five RC extracts can effectively bind Fe^2+^ ion, suggests that metal-chelating may be one of mechanisms for scavenging ·OH or ·O_2_^-^.

In order to verify whether five RC extracts can directly scavenge radicals. We determined their DPPH· and ABTS·^+^ radical-scavenging abilities.

**Scheme 3:**
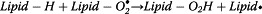
The proposal reaction of caffeic acid to scavenge DPPH· (step 1).

As seen in Table 1 and Additional file 2, five RC extracts scavenged both DPPH· and ABTS·^+^ radicals in a dose-dependent manner. The previous studies have demonstrated that DPPH · may be scavenged by an antioxidant through donation of hydrogen atom (H·) to form a stable DPPH-H molecule [16-18]. Caffeic acid, a phenolic acid occurring in RC, for example, could scavenge DPPH· via the proposed reaction (Scheme 3) [19].

**Scheme 4:**
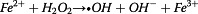
The proposal reaction of caffeic acid to scavenge DPPH· (step 2).

Radical (A) can be further withdrawn hydrogen atom (H·) to form stable quinone (B) (Scheme 4).

**Scheme 5:**
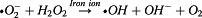
The proposal reaction of caffeic acid and ABTS ·^+^.

Unlike DPPH· scavenging, ABTS ·^+^ scavenging however is considered as an electron (e) transfer reaction [20]. For example, the proposed reaction for caffeic acid to scavenge ABTS ·^+^ can be briefly illustrated using Scheme 5. Similarly, if ABTS ·^+^ is excessive, radical (A) can also further change to quinone (B).

As illustrated in Scheme 3, 4 and 5, the generations of both DPPH· and ABTS·^+^radicals did not rely on the transition metal catalysis.

Based on the discussion above, it can be deduced that direct radical-scavenging was regarded as another mechanism for five RC extract to scavenge ·OH or ·O_2_^-^, and they exerted radical-scavenging action by donating hydrogen atom (H·) and electron (e).

Until now, about 200 compounds have been found in RC [21]. Generally, they belong to three different classes, i.e. phenolics, saponins, and polysaccharides. In the study, we used chemical method to measure the contents of total phenolics, total saponins, and total sugars in fiver RC extracts (Table 2). On the other hand, since 1/IC_50_ value showed parallelism with antioxidant level, it was therefore used to evaluate antioxidant level in the study (Additional file 2). On this basis, forty-eight correlation graphs were plotted to quantitative analyze the correlation between antioxidant levels and chemical contents in five RC extracts (Additional file 3). The correlation coefficients (R values) in Table 3 indicated that, total phenolics were of significant positive correlations (R = 0.17 ~ 0.99; average, 0.56) with antioxidant levels, while total sugars and total saponins had no positive correlations (for total sugars, R = −0.88 ~ 0.46, the average was −0.20; for total saponins, R = −0.92 ~ 0.94, the average was −0.26) with antioxidant levels. The data clearly demonstrated the significant contribution of phenolic compounds to the antioxidant of RC.

**Table 2:** Chemical contents of five RC extracts

**Extracts**	**PERC**	**EARC**	**AERC**	**95ERC**	**WRC**
Total phenolics (mg Pyr./g)	13.17 ± 0.43^a^	65.01 ± 0.34 ^d^	15.73 ± 0.34 ^b^	31.10 ± 1.30 ^c^	12.46 ± 1.00 ^a^
Total sugars (mg Glucose/g)	237.16 ± 38.67 ^a^	175.46 ± 21.50 ^a^	624.75 ± 17.62 ^b^	603.48 ± 18.74 ^b^	804.54 ± 50.53 ^c^
Total saponins (mg Ole./g)	917.52 ± 87.83 ^c^	379.46 ± 15.46 ^b^	185.16 ± 4.10 ^a^	180.44 ± 1.95 ^a^	198.34 ± 12.30 ^a^
Caffeic acid (peak area)	2.96 ± 0.85 ^a^	125.22 ± 8.84 ^c^	6.45 ± 2.93 ^b^	14.19 ± 10.14 ^b^	2.79 ± 1.23 ^a^
Ferulic acid (peak area)	0.89 ± 0.44 ^c^	79.31 ± 3.22 ^d^	0.39 ± 0.37 ^b^	0.68 ± 1.18 ^c^	0.0040 ± 0.0010 ^a^
Isoferulic acid (peak area)	4.92 ± 1.68 ^a^	33.24 ± 0.44 ^d^	12.77 ± 2.56 ^c^	8.68 ± 0.28 ^b^	--

Each value is expressed as mean ± SD (*n* = 3). Means values with different superscripts in the same row are significantly different (*p <* 0.05), while with same superscripts are not signifiacntly different (*p >* 0.05)**.***PERC*, petroleum ether extract of rhizoma *Cimicifugae; EARC*, ethyl acetate extract of rhizoma *Cimicifugae; AERC*, absolute ethanol extract of rhizoma *Cimicifugae; 95ERC*, 95% ethanol extract of rhizoma *Cimicifugae; WRC*, water extract of rhizoma *Cimicifugae.* --: Below the detection limit. Pyr., pyrogallo; Ole., Oleanic acid.

**Table 3:** **The R values between chemical contents and 1/IC**_**50**_

	**Total phenolics**	**Caffeic acid**	**Ferulic acid**	**Isoferulic acid**	**Total sugars**	**Total saponins**
Anti-LPO	0.99	0.98	0.96	0.95	−0.69	−0.001
DNA	0.61	0.38	0.37	0.40	−0.11	−0.35
·OH	0.57	0.37	0.34	0.47	0.11	−0.68
·O_2_^-^	0.36	0.38	0.33	0.53	−0.53	0.14
Chelating	0.17	0.31	0.31	0.21	−0.88	0.94
DPPH·	0.43	0.39	0.35	0.35	0.46	−0.92
ABTS·^+^	0.57	0.54	0.61	0.49	0.30	−0.84
Reducing	0.82	0.65	0.68	0.68	−0.26	−0.39
**Average**	**0.56**	**0.51**	**0.50**	**0.51**	**−0.20**	**−0.26**

R, correlation coefficient; 1/IC_50_: the unit is mL/μg, all 1/IC_50_ values are listed in Additional file 2. LPO, lipid peroxidation.

**Figure 1:**
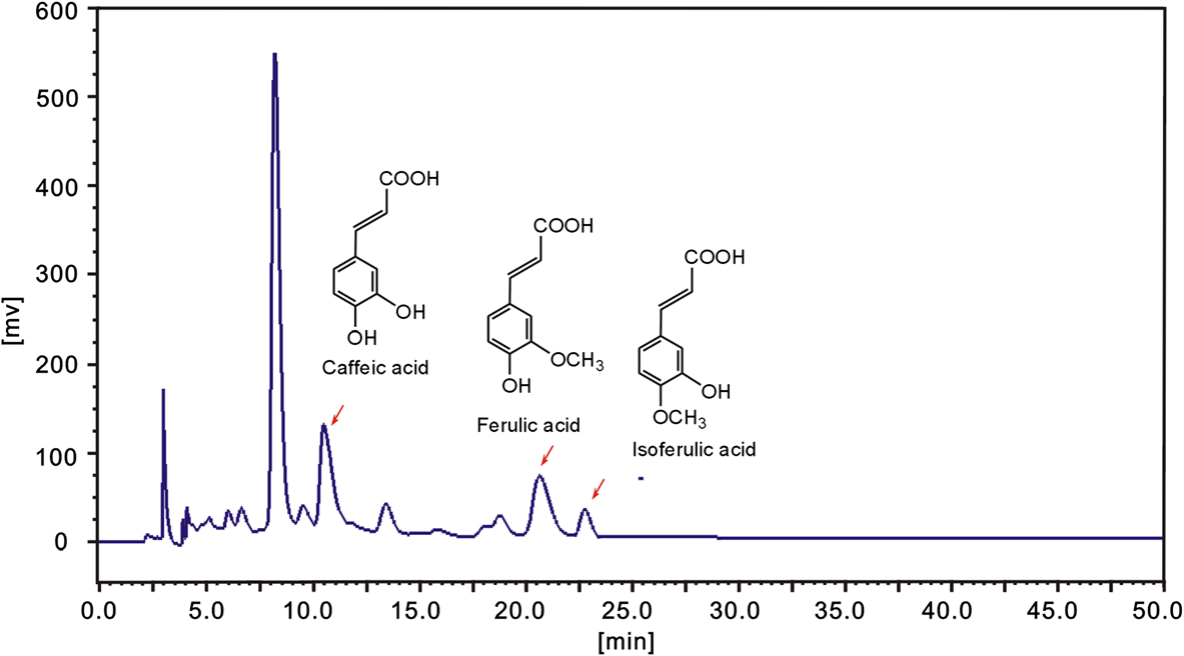
**A typical HPLC profile of EARC (ethyl acetate extract of rhizoma *Cimicifugae*).** Syltech P510 HPLC system (Los Angeles, California, USA), Dikma Diamonsil C_18_ (250 mm × 4.6 mm, 5 μm size) (Beijing, China), acetonitrile-0.5% acetic acid in water (17:83, v: v), 1.0 mL/min flow rate, 30 μL injection, 316 nm wavelength.

Among total phenolics, several compounds had been isolated from RC, including salicylic acid, caffeic acid, ferulic acid, isoferulic acid, flavonoids and tannins. In our study, however, three phenolic acids caffeic acid, ferulic acid and isoferulic acid were also identified by HPLC assay (Figure 1 and Additional file 4), in which peak areas were used to characterize the relative contents of three phenolic acids.

Then, we used the peak areas to plot the correlation graphs *vs* 1/IC_50_ values of five RC extracts, to obtain the correlation coefficients (R values). As shown in Additional file 5 and Table 3, three phenolic acids all presented significant positive correlations (average *R* value = 0.47, 0.44, and 0.51, respectively for caffeic acid, ferulic acid and isoferulic acid). On the other hand, previous works have shown that three phenolic acids possess strong antioxidant ability [22-24]. Hence, they are considered as three of main bioactive compounds relevant to antioxidant in RC.

### Experimental

#### Chemicals and plant material

Trolox (± − 6-hydroxyl-2,5,7,8-tetramethlyhromane-2-carboxylic acid), Ferrozin [3-(2-pyridyl)-5,6-bis (4-phenylsulfonicacid)-1,2,4-triazine], DPPH · (1,1-Diphenyl-2-picrylhydrazyl radical), pyrogallol, linoleic acid, BHT (2.6-ditertiary butyl-p-cresol) and murexide (5,5^′^-Nitrilodibarbituric acid monoammonium salt) were purchased from Sigma Co. (Sigmaaldrich Trading Co., Shanghai, China); ABTS diammonium salt [2,2^′^-Azino-bis (3-ethylbenzothiazoline-6-sulfonic acid diammonium salt)], D-2-deoxyribose, and GSH (glutathione) were Amresco Inc. (Solon, OH, USA); DNA sodium salt (fish sperm) was purchased from Aladdin Chemistry Co. (Shanghai, China); Ferulic acid, caffeic acid were purchased from National Institute for the Control of Pharmaceutical and Biological Products (Beijing, China); Acetonitrile, methanol and water were of HPLC grade; All other chemicals used were in analytical grade.

Rhizoma *Cimicifugae* was purchased from Guangzhou University of Chinese Medicine Yanghe Interlink Limited Company and identified by Prof. Shuhui Tan. A voucher specimen was deposited in our laboratory.

#### Preparation of different extracts *of* rhizoma *Cimicifugae*

**Figure 2:**
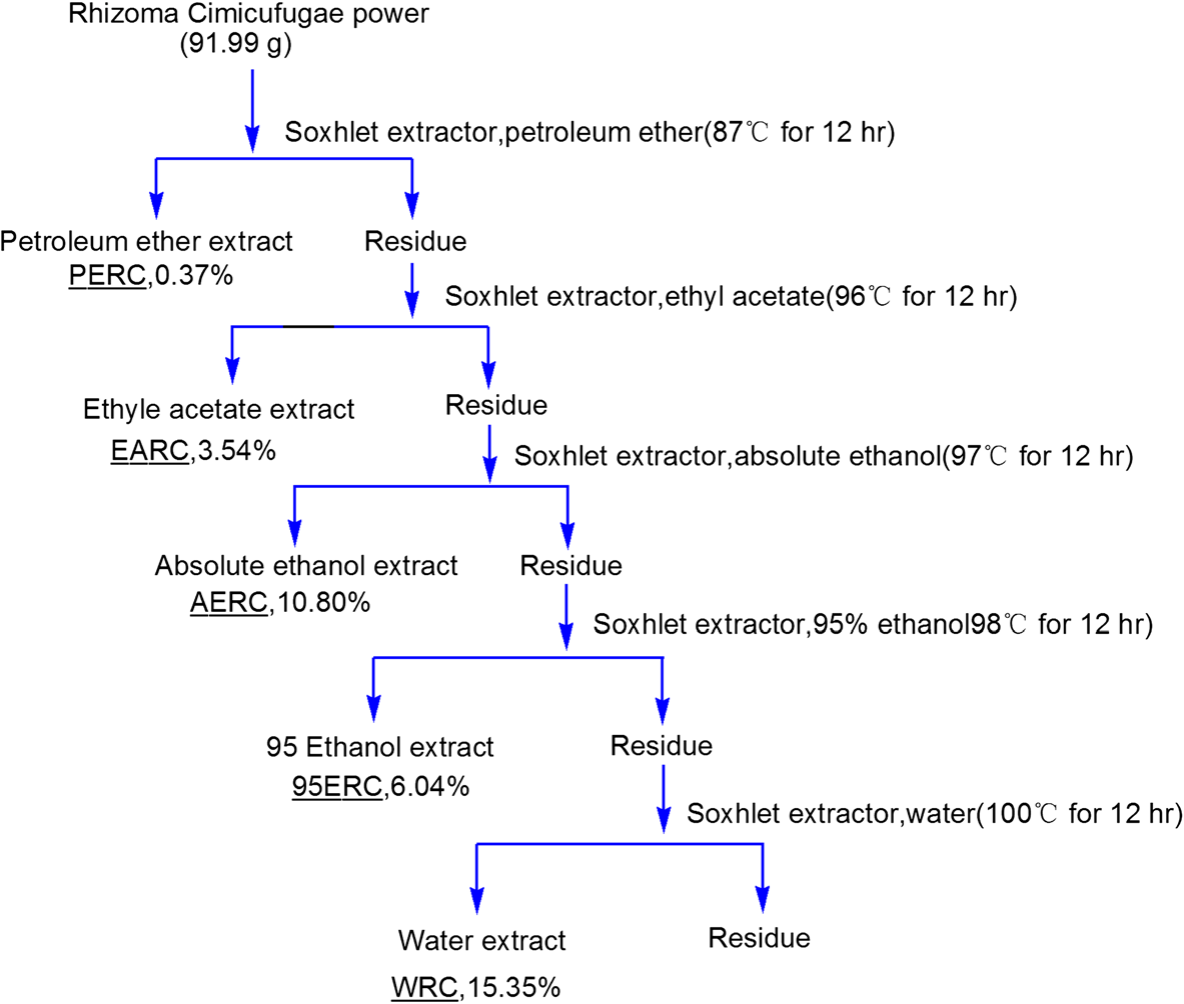
**Preparation of five extracts of rhizoma *Cimicifugae***.

The dried rhizoma *Cimicifugae* was coarsely powder then extracted in sequence with petroleum ether (60–90), ethyl acetate, ethanol, 95% ethanol and water by Soxhlet extractor for 12 hours. The extracts were concentrated under reduced pressure to a constant weight. Then the dried extracts were stored at 4°C until used (Figure 2).

#### Protective effect against lipid-peroxidation

The protective effect against lipid-peroxidation was investigated using a linoleic acid emulsion [25]. The linoleic acid emulsion was prepared by mixing and homogenizing 312.6 mg of linoleic acid, 78.2 mg of Tween-20 as emulsifier, and 30 mL of 75% ethanol (v/v), 0.1 mL of various concentrations of samples (0.4-2.8 mg/mL) were added to 1.5 mL of linoleic acid emulsion and 0.4 mL distilled water. The reaction mixture (2 mL) was incubated at room temperature in glass bottles for 72 hours. To 0.15 mL of sample solution, 3.65 mL of 75% ethanol, 0.1 mL of ammonium thiocyanate (30%, m/v), and 0.1 mL of ferrous chloride (0.02 M in 3.6% HCl) were added. The mixture was diluted to two-fold volume with methanol in case the color was too dark, the peroxide value was measured by monitoring absorbance at 500 nm in a spectrophotometer (Unico 2100, Shanghai, China). The percentage of inhibition of lipid-peroxidation in linoleic acid emulsion was calculated by following equation:

(6) $$\;\mathrm{Inhibition}\;\%=\frac{A_{0}-A}{A_{0}}\times 100\%$$

Where *A* is the absorbance with samples, while *A*_*0*_ is the absorbance without samples.

#### Protective effect against hydroxyl-induced DNA damage

The experiment was conducted as described in previous report [26]. However, deoxyribose was replaced by DNA sodium salt. Briefly, sample was dissolved in methanol to prepare the sample solution. Various amounts (10–100 μL) of sample solutions (10 mg/mL) were then separately taken into mini tubes. After evaporating the sample solution in tube to dryness, 400 μL phosphate buffer (0.2 M, pH 7.4) was brought to the sample residue. Then, 50 μL DNA (10.0 mg/mL), 75 μL H_2_O_2_ (33.6 mM), 50 μL FeCl_3_ (0.3 mM) and 100 μL Na_2_EDTA solutions (0.5 mM) were added. The reaction was initiated by mixing 75 μL ascorbic acid (1.2 mM) . After incubation in a water bath at 55°C for 20 min, the reaction was terminated by 250 μL trichloroacetic acid (0.6 M). The color was then developed by addition of 150 μL 2-thiobarbituric acid (TBA) (0.4 M, in 1.25% NaOH aqueous solution) and heated in an oven at 105°C for 15 min. The mixture was cooled and absorbance was measured at 530 nm against the buffer (as blank). The percent of protection of DNA is expressed as follows:

(7) $$\;\mathrm{Protective}\;\mathrm{effect}\;\%=\frac{A_{0}-A}{A_{0}}\times 100\%$$

Where *A* is the absorbance with samples, while *A*_*0*_ is the absorbance without samples.

#### Hydroxyl (·OH) radical-scavenging assay

The hydroxyl radical-scavenging activity was investigated by the deoxyribose degradation method [26], with some modifications. In brief, the sample was dissolved in methanol, and then the sample solution was aliquoted into mini tubes. After evaporating the sample solutions in the tubes to dryness (48–240 μg), 300 μL of phosphate buffer (0.2 M, pH 7.4) was added to the sample residue. Subsequently, 50 μL deoxyribose (2.8 mM), 50 μL H_2_O_2_ (2.8 mM), 50 μL FeCl_3_ (25 μM), and 100 μL Na_2_EDTA (0.8 mM) were added. The reaction was initiated by mixing 50 μL ascorbic acid (1.2 mM) and the total volume of the reaction mixture was adjusted to 600 μL with buffer. After incubation in a water bath at 50°C for 20 min, the reaction was terminated by addition of 500 μL trichloroacetic acid (5%, w/w). The color was then developed by addition of 500 μL TBA (1 g/100 mL, in 1.25% NaOH aqueous solution) and heated in an oven at 105°C for 15 min. The mixture was cooled and the absorbance was measured at 532 nm against the buffer (as a blank control). The inhibition percentage for ·OH was expressed as follows:

(8) $$\;\mathrm{Inhibition}\;\%=\frac{A_{0}-A}{A_{0}}\times 100\%$$

Where *A* is the absorbance containing samples, while *A*_*0*_ is the absorbance without samples.

#### Superoxide anion (·O_2_^-^) radical-scavenging assay

Measurement of superoxide anion (·O_2_^−^) scavenging activity was based on our method [27]. Briefly, samples were dissolved in methanol at 3 mg/mL. The sample solution *x* μL (*x* = 67, 117, 167, 217, 267, and 317) was mixed with Tris–HCl buffer (2950 - *x* μL, 0.05 M, pH 8.2) containing EDTA (1 mM) and pyrogallol (50 μL, 6 mM in 10.0 mM HCl), then shaken rapidly at 37°C. The absorbance at 325 nm of the mixture was measured (Unico 2100, Shanghai, China) against the Tris–HCl buffer as blank every 30 s for 5 min. The slope of the correlation of absorbance with time was calculated. The reaction mixture without sample was used as the control. The ·O_2_^-^ scavenging ability was calculated as:

(9) $$\;\left(\frac{\Delta A_{325\mathrm{nm},\mathrm{control}}}{T}-\frac{\Delta A_{325\mathrm{nm},\mathrm{sample}}}{T}\right)/\frac{\Delta A_{325\mathrm{nm},\mathrm{control}}}{T}\times \;100\%\;$$

Here, *ΔA*_*325nm, control*_ is the increase in *A*_*325nm*_ of the mixture without the sample and *ΔA*_*325nm, sample*_ is that for the mixture with the sample; *T* = 5 min. The experiment temperature was 37°C.

#### Chelating activity on Cu^2+^

The Cu^2+^-chelating activities of five RC extracts were measured by the method [28]. Briefly, 60 μL CuSO_4_ aqueous solution (20 mM) was added to hexamine HCl buffer (pH 5.3, 30 mM) containing 30 mM KCl and 0.20 mM murexide. After incubation for 1 min at room temperature, 80–230 μL sample solutions (4 mg/mL in methanol) were added. The final volume was adjusted to 1500 μL with methanol. Then, the mixture was shaken vigorously and left at room temperature for 10 min. Absorbance of the solution was then measured by a spectrophotometer (Unico 2100, Shanghai, China) at 485 nm and 520 nm. The absorbance ratio (*A*_*485*_*/A*_*520*_) reflected the free Cu^2+^ content. Therefore, the percentage of cupric chelating effect was calculated by the following formula:

(10) $$\;\mathrm{Relative}\;\mathrm{chelating}\;\mathrm{effect}\;\%=\frac{{\left(\frac{A_{485}}{A_{520}}\right)}_{\max}-\left(\frac{A_{485}}{A_{520}}\right)}{{\left(\frac{A_{485}}{A_{520}}\right)}_{\max}-{\left(\frac{A_{485}}{A_{520}}\right)}_{\min}}\times 100\%$$

Where $\left(\frac{A_{485}}{A_{520}}\right)$ is the absorbance ratio of the sample, while ${\left(\frac{A_{485}}{A_{520}}\right)}_{\max}$ is the maximum absorbance ratio and ${\left(\frac{A_{485}}{A_{520}}\right)}_{\min}$ is the minimum absorbance ratio in the test.

#### DPPH· scavenging assay

DPPH· radical-scavenging activity was determined as previously described by Li [23]. Briefly, 0.8 mL DPPH· solution (0.1 M) was mixed with 4.2 mL various concentrations (15–300 μg/mL) of samples dissolved in 95% ethanol. The mixture was kept at room temperature for 30 min, and then measured with a spectrophotometer (Unico 2100, Shanghai, China) at 519 nm. The DPPH· inhibition percentage of the samples was calculated:

(11) $$\;\mathrm{Inhibition}\;\%=\frac{A_{0}-A}{A_{0}}\times 100\%$$

Where *A*_*0*_ is the absorbance without samples, while *A* is the absorbance with samples.

#### ABTS·^+^ scavenging assay

The ABTS·^+^-scavenging activity was measured as described [23] with some modifications. The ABTS·^+^ was produced by mixing 0.35 mL ABTS diammonium salt (7.4 mM) with 0.35 mL potassium persulfate (2.6 mM). The mixture was kept in the dark at room temperature for 12 hours to allow completion of radical generation, then diluted with 95% ethanol (about 1:50) so that its absorbance at 734 nm was 0.70 ± 0.02. To determine the scavenging activity, 1.2 mL aliquot of ABTS·^+^ reagent was mixed with 0.3 mL of sample ethanolic solutions (40–540 μg/mL). After incubation for 6 min, the absorbance at 734 nm was read on a spectrophotometer (Unico 2100, Shanghai, China). The percentage inhibition of the samples was calculated as:

(12) $$\;\mathrm{Inhibition}\;\%=\frac{A_{0}-A}{A_{0}}\times 100\%$$

Where *A*_*0*_ is the absorbance at 734 nm without samples, while *A* is the absorbance at 734 nm with samples.

#### Reducing power (Fe^3+^) assay

Ferric cyanide (Fe^3+^) reducing power was determined according to the method of Oyaizu [23,29]. In brief, sample solutions *x* μL (4 mg/mL, *x* = 20, 40, 60, 80, and 100) were mixed with (350-*x*) μL Na_2_HPO_4_/KH_2_PO_4_ buffer (0.2 M, pH 6.6) and 250 μL K_3_Fe(CN)_6_ aqueous solution (1 g/100 mL).

The mixture was incubated at 50°C for 20 min, 250 μL of trichloroacetic acid (10 g/100 mL) was added, and the mixture was centrifuged at 3500 r/min for 10 min. As soon as 400 μL supernatant was mixed with 400 μL FeCl_3_ (0.1 g/100 mL in distilled water), the timer was started. At 90 s, absorbance of the mixture was read at 700 nm (Unico 2100, Shanghai, China). Samples were analyzed in groups of three, and when the analysis of one group has finished, the next group of three samples were mixed with FeCl_3_ to prevent the mixture from being oxidized by air. The relative reducing ability of the sample was calculated by using the formula:

(13) $$\;\mathrm{Relative}\;\mathrm{reducing}\;\mathrm{effect}\%=\frac{A-A_{\min}}{A_{\max}-A_{\min}}\times 100\%$$

Here, *A*_*max*_ is the maximum absorbance and *A*_*min*_ is the minimum absorbance in the test. *A* is the absorbance of sample.

#### HPLC analysis for caffeic acid, ferulic acid, and isoferulic acid

Caffeic acid, ferulic acid, and isoferulic acid were identified by comparing their retention times using HPLC method. HPLC analysis was performed on a Syltech P510 system (Los Angeles, California, USA) equipped with Dikma Diamonsil C_18_ (250 mm × 4.6 mm, 5 μm) (Beijing, China). The mobile phase consisted of acetonitrile-0.5% acetic acid in water (17:83, v: v), the flow rate was 1.0 mL/min, injection volume was 30 μL and absorption was measured at 316 nm.

#### Determination of total phenolics, total sugars and total saponins

The total phenolics of five RC extracts were determined by the Folin-Ciocalteu method with a little modifications [30]. Firstly, 0.5 mL extract methanolic solution ( 0.4 mg/mL ) was mixed with 0.5 mL 0.25 M Folin-Ciocalteu reagent. After incubation for 3 min, 1 mL of Na_2_CO_3_ solution (15%, w/v) was added. After standing at the room temperature for 30 min, the mixture was centrifuged at 3500 r/min for 3 min. The absorbance of the supernatant was measured at 760 nm (Unico 2100, Shanghai, China). The determinations were performed in triplicate, and the calculations were based on a calibration curve obtained with pyrogallol. The result was expressed as pyrogallol equivalents (Pyr.) in milligrams per gram of extract.

The total sugars were evaluated according to the phenol-sulfuric acid method [30]. A 40-μL aliquot of sample solution (1 mg/mL) was placed in a flask, then 210 μL distilled water, 250 μL phenol solution (5%, w/v) and 250 μL concentrated sulfuric acid were added. After incubation for 20 min at room temperature, the absorbance of reaction mixture was measured at 490 nm (Unico 2100, Shanghai, China). The measurements were performed in triplicate, and the calculations were based on a calibration curve obtained with glucose. The result was expressed as glucose equivalents in milligrams per gram of extract.

The total saponins were measured according to the method [30]. RC extract was dissolved in methanol to prepare the sample solution (4 mg/mL), then a 20-μL aliquot of sample solution was taken into a mini tube. After the sample solution in tube was evaporated to dryness (water bath, 80°C), 0.1 mL vanillin-acetic acid solution (5 mg/mL) and 0.4 mL perchloric acid were added to the sample residue. The reaction mixture was incubated at 70°C for 15 min, then diluted by 1.25 mL acetic acid. The mixture was measured using a spectrophotometer (Unico 2100, Shanghai, China) at 540 nm against a blank control, which contained all reagents except sample. All analyses were run in triplicate. Quantification was based on the standard curve for oleanic acid (10–79 μg/mL) and the results were expressed in milligrams of oleanic acid (Ole.) equivalents per gram of extract.

#### The correlation graphs and R values

In order to investigate the correlation between antioxidant activities and chemical components of five RC extracts, 48 correlation graphs were plotted between 1/IC_50_ values (including of LPO, DNA, ·OH, ·O_2_^-^ ,Cu-chelating, DPPH·, ABTS·^+^, and reducing power assays) and chemical contents (including total phenolics, caffeic acid, ferulic acid, isoferulic acid, total sugars, and total saponins). In the correlation graphs, the correlation coefficients (R values) were calculated by Origin 6.0 professional software.

#### Statistical analysis

Data are given as the mean ± SD of three measurements. The IC_50_ values were calculated by linear regression analysis. All linear regression in this paper was analyzed by Origin 6.0 professional software. Significant differences were performed using the *T*-test (*p <* 0.05). The analysis was performed using SPSS software (v.12, SPSS, USA).

## Conclusion

As an effective antioxidant, rhizoma *Cimicifugae* can protect DNA and lipids against oxidative damage. Its antioxidant ability can be responsible for its various pharmacological effects and may be mainly attributed to the existence of total phenolics, among which caffeic acid, ferulic acid and isoferulic acid are regarded as main bioactive components. Rhizoma *Cimicifugae* exerts its antioxidant effect through metal-chelating, and radical-scavenging which is via donating hydrogen atom (H·) and donating electron (e).

## Abbreviations

RC: Rhizoma *Cimicifugae*; PERC: Petroleum ether extract from rhizoma *Cimicifugae*; EARC: Ethyl acetate extract from rhizoma Cimicifugae; AERC: Absolute ethanol extract from rhizoma *Cimicifugae*; 95ERC: 95% ethanol extract from rhizoma *Cimicifugae*; WRC: Water extract from rhizoma *Cimicifugae*; DPPH·: 1,1-diphenyl-2-picryl-hydrazl radical; ABTS: 2,2′-azino-bis (3-ethylbenzo- thiazoline-6-sulfonic acid salt); ROS: Reactive oxygen species; TCM: Traditional Chinese medicine; BHT: 2,6-di-tert-butyl-4-methyphenol; GSH: Glutathione; BHA: Butylated hydroxyanisole; TBARS: Thiobarbituric acid reactive substances; TBA: Thiobarbituric acid; MDA: Malondialdehyde.

## Competing interests

The authors declare that they have no competing interests.

## Authors’ contribution

XL & JL made a significant contribution to experiment design, acquisition of data, analysis, drafting of the manuscript. YG and WH have made a substantial contribution to interpretation of data, drafting. DC has carefully revised the manuscript for intellectual content. All authors read and approved the final manuscript.

## Supplementary Material

Additional file 1 Presents the photo of rhizoma *Cimicifugae.* Click here for file

Additional file 2 Contain all dose response curves, IC_50_, and 1/C_50_ values of antioxidant assays. Click here for file

Additional file 3 Provides the calculations of contents of total phenolics, total saponins, and total sugars. Click here for file

Additional file 4 Shows HPLC figures and peak areas. Click here for file

Additional file 5 Includes all correlation graphs. Click here for file
