# Supplementary material for: Antioxidant activity and mechanism of Rhizoma Cimicifugae
Source: Chem Cent J. 2012 Nov 23;6:140. doi: 10.1186/1752-153X-6-140 (PMC3557226; doi:10.1186/1752-153X-6-140)
Supplement: Additional file 1 — Presents the photo of rhizoma Cimicifugae. [file 1752-153X-6-140-S1.doc]

Additional file 1-Photo

**Antioxidant Activity and Mechanism of rhizoma *Cimicifugae***

Xican Li^‡,1^, Jing Lin^‡,1^, Yaoxiang Gao^1^, Weijuan Han^1^, and Dongfeng Chen*^,2^

^1^*School of Chinese Herbal Medicine,* ^2^*School of Basic medicine, Guangzhou University of Chinese Medicine, Guangzhou, 510006, China*

^*^Corresponding author: CDF27212@21cn.com

^‡^ Both authors contributed equally to this work.

----------------------------------------------------------------------------------------------------------------------


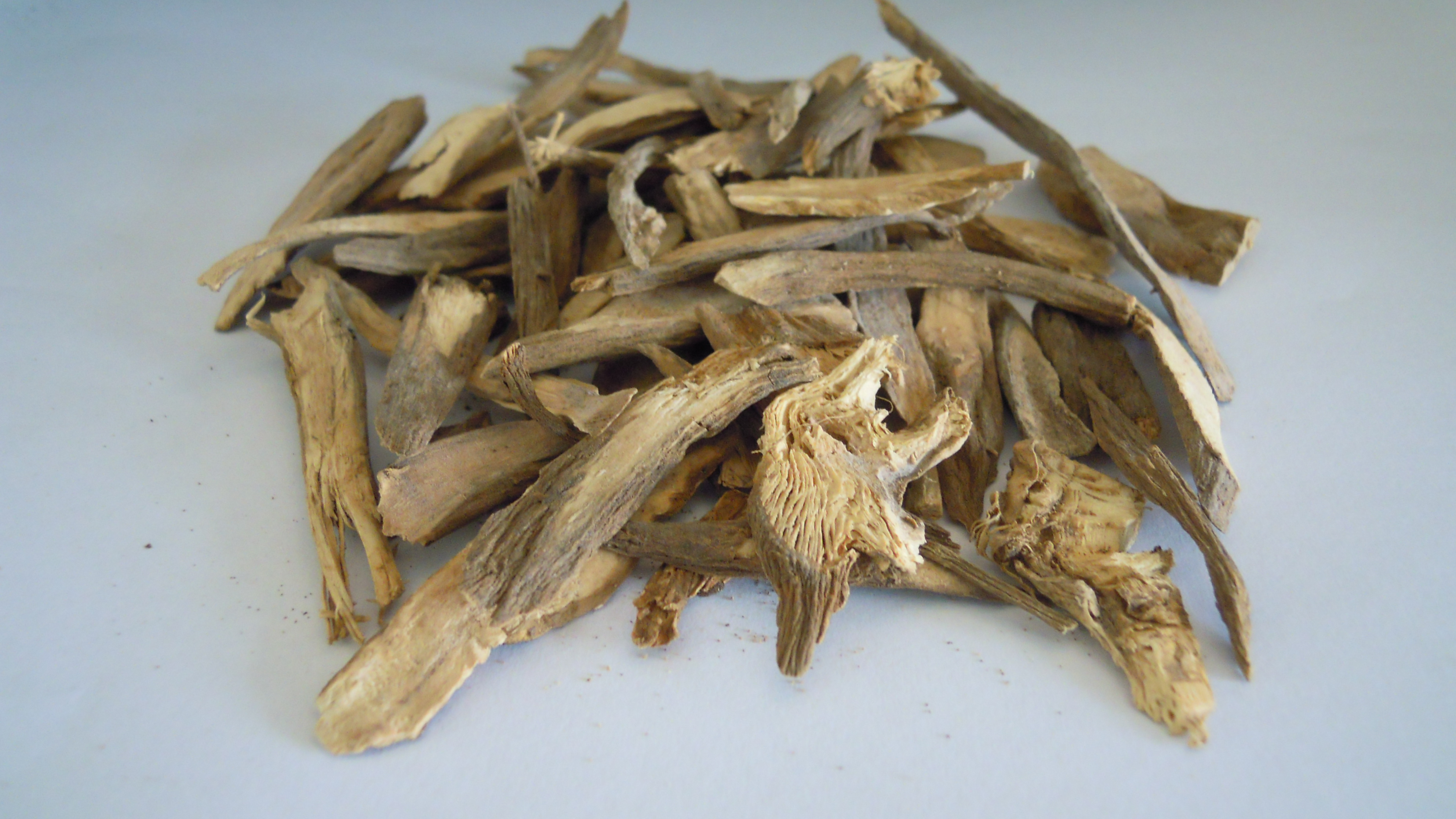


Figure A1.1 Dried rhizoma *Cimicifugae*

Contributed by Jing Lin. (Guangzhou, Sept., 2012)
