# Supplementary material for: Antioxidant activity and mechanism of Rhizoma Cimicifugae
Source: Chem Cent J. 2012 Nov 23;6:140. doi: 10.1186/1752-153X-6-140 (PMC3557226; doi:10.1186/1752-153X-6-140)
Supplement: Additional file 2 — Contain all dose response curves, IC50, and 1/C50 values of antioxidant assays. [file 1752-153X-6-140-S2.doc]

**Additional file 2- Dose response curves**

**Antioxidant Activity and Mechanism of rhizoma *Cimicifugae***

Xican Li*^,‡,1^, Jing Lin^‡,1^, Yaoxiang Gao^1^, Weijuan Han^1^, and Dongfeng Chen*^,2^

^1^*School of Chinese Herbal Medicine,* ^2^*School of Basic Medical Science, Guangzhou University of Chinese Medicine, Guangzhou, 510006, China*

^*^Corresponding author: lixican@126.com;cdf27212@21cn.com

^‡^ Both authors contributed equally to this work.

----------------------------------------------------------------------------------------------------------------------

**Abstract**

The Additional file 2 provides dose response curves, IC_50_ and 1/C_50_ values of all antioxidant assays.

The data **underlined** are cited by the main text.

**Figure A2.**1 The dose response curves of anti-lipid peroxidation. Each value is expressed as mean ± standard deviation, n =3. PERC: petroleum ether extract from rhizoma *Cimicifugae*; EARC:ethyl acetate extract from rhizoma Cimicifugae; AERC: absolute ethanol extract from rhizoma *Cimicifugae*; 95ERC: 95% ethanol extract from rhizoma *Cimicifugae*; WRC: water extract from rhizoma *Cimicifugae*.

**Table A2.1** The IC_50_ and 1/IC_50_ values for anti-lipid peroxidation

|  | PERC | EARC | AERC | 95ERC | WRC | BHA |
| --- | --- | --- | --- | --- | --- | --- |
| IC_50_ (μg/mL) | 13.99±2.11^d^ | 4.55±0.44 ^b^ | 14.92±4.31 ^e^ | 10.62±0.97 ^c^ | 19.12±2.04^f^ | 0.028±0.00 ^a^ |
| 1/IC_50_ (mL/μg) | 0.0715 | 0.220 | 0.0670 | 0.0942 | 0.0523 |  |

IC_50_ value is defined as the concentration of 50% protection percentage. It was calculated by linear regression analysis and expressed as Mean±SD (n=3). The linear regression was analyzed by Origin 6.0 professional software. Means values with different superscripts in the same row are significantly different (*p<*0.05); Means values with same superscripts in the same row are not significantly different (*p<*0.05). PERC: petroleum ether extract from rhizoma *Cimicifugae*; EARC:ethyl acetate extract from rhizoma Cimicifugae; AERC: absolute ethanol extract from rhizoma *Cimicifugae*; 95ERC: 95% ethanol extract from rhizoma *Cimicifugae*; WRC: water extract from rhizoma *Cimicifugae*.

**Figure A2.2** The dose response curves of DNA protective effect. Each value is expressed as mean ± standard deviation, n =3. PERC: petroleum ether extract from rhizoma *Cimicifugae*; EARC:ethyl acetate extract from rhizoma Cimicifugae; AERC: absolute ethanol extract from rhizoma *Cimicifugae*; 95ERC: 95% ethanol extract from rhizoma *Cimicifugae*; WRC: water extract from rhizoma *Cimicifugae*.

**Table A2.2** The IC_50_ and 1/IC_50_ values for DNA protective effect

|  | PERC | EARC | AERC | 95ERC | WRC | Trolox |
| --- | --- | --- | --- | --- | --- | --- |
| IC_50_ (μg/mL) | ND | 1905.51±177.54 ^c^ | 14397.18±1075.01 ^d^ | 1090.11±21.25 ^b^ | ND | 144.42±2.66 ^a^ |
| 1/IC_50_ (mL/μg) | 0 | 0.000525 | 0.0000695 | 0.000917 | 0 |  |

IC_50_ value is defined as the concentration of 50% protection percentage. It was calculated by linear regression analysis and expressed as Mean±SD (n=3). The linear regression was analyzed by Origin 6.0 professional software. Means values with different superscripts in the same row are significantly different (*p<*0.05); Means values with same superscripts in the same row are not significantly different (*p<*0.05). ND: too large to be measured. PERC: petroleum ether extract from rhizoma *Cimicifugae*; EARC:ethyl acetate extract from rhizoma Cimicifugae; AERC: absolute ethanol extract from rhizoma *Cimicifugae*; 95ERC: 95% ethanol extract from rhizoma *Cimicifugae*; WRC: water extract from rhizoma *Cimicifugae*.

**Figure A2.3** The dose response curves of •OH scavenging activity. Each value is expressed as mean ± standard deviation, n =3. PERC: petroleum ether extract from rhizoma *Cimicifugae*; EARC:ethyl acetate extract from rhizoma Cimicifugae; AERC: absolute ethanol extract from rhizoma *Cimicifugae*; 95ERC: 95% ethanol extract from rhizoma *Cimicifugae*; WRC: water extract from rhizoma *Cimicifugae*.

**Table A2.3** The IC_50_ and 1/IC_50_ values for •OH scavenging activity

|  | PERC | EARC | AERC | 95ERC | WRC | Trolox |
| --- | --- | --- | --- | --- | --- | --- |
| IC_50_ (μg/mL) | 561.19±21.16 ^f^ | 111.55±1.32 ^c^ | 132.32±0.15 ^d^ | 84.30±1.02 ^b^ | 233.45±15.70 ^e^ | 37.67±0.67 ^a^ |
| 1/IC_50_ (mL/μg) | 0.00178 | 0.00896 | 0.00756 | 0.0119 | 0.00428 |  |

IC_50_ value is defined as the concentration of 50% protection percentage. It was calculated by linear regression analysis and expressed as Mean±SD (n=3). The linear regression was analyzed by Origin 6.0 professional software. Means values with different superscripts in the same row are significantly different (*p<*0.05); Means values with same superscripts in the same row are not significantly different (*p<*0.05). PERC: petroleum ether extract from rhizoma *Cimicifugae*; EARC:ethyl acetate extract from rhizoma Cimicifugae; AERC: absolute ethanol extract from rhizoma *Cimicifugae*; 95ERC: 95% ethanol extract from rhizoma *Cimicifugae*; WRC: water extract from rhizoma *Cimicifugae*.

**Figure A2.4** The dose response curves of •O_2_^-^ scavenging activity. Each value is expressed as mean ± standard deviation, n =3. PERC: petroleum ether extract from rhizoma *Cimicifugae*; EARC:ethyl acetate extract from rhizoma Cimicifugae; AERC: absolute ethanol extract from rhizoma *Cimicifugae*; 95ERC: 95% ethanol extract from rhizoma *Cimicifugae*; WRC: water extract from rhizoma *Cimicifugae*.

**Table A2.4** The IC_50_ and 1/IC_50_ values for •O_2_^-^ scavenging activity

|  | PERC | EARC | AERC | 95ERC | WRC | GSH |
| --- | --- | --- | --- | --- | --- | --- |
| IC_50_ (μg/mL) | 322.81±14.90 ^b^ | 306.68±0.87 ^b^ | 284.36±11.43 ^b^ | 301.08±8.34 ^b^ | 650.65±59.55 ^c^ | 81.65±5.21 ^a^ |
| 1/IC_50_ (mL/μg) | 0.0031 | 0.0033 | 0.0035 | 0.0033 | 0.0015 |  |

IC_50_ value is defined as the concentration of 50% protection percentage. It was calculated by linear regression analysis and expressed as Mean±SD (n=3). The linear regression was analyzed by Origin 6.0 professional software. Means values with different superscripts in the same row are significantly different (*p<*0.05); Means values with same superscripts in the same row are not significantly different (*p<*0.05). PERC: petroleum ether extract from rhizoma *Cimicifugae*; EARC:ethyl acetate extract from rhizoma Cimicifugae; AERC: absolute ethanol extract from rhizoma *Cimicifugae*; 95ERC: 95% ethanol extract from rhizoma *Cimicifugae*; WRC: water extract from rhizoma *Cimicifugae*.

**Figure A2.5** The dose response curves of Cu^2+^ chelating ability assay.Each value is expressed as mean ± standard deviation, n =3. PERC: petroleum ether extract from rhizoma *Cimicifugae*; EARC:ethyl acetate extract from rhizoma Cimicifugae; AERC: absolute ethanol extract from rhizoma *Cimicifugae*; 95ERC: 95% ethanol extract from rhizoma *Cimicifugae*; WRC: water extract from rhizoma *Cimicifugae*.

**Table A2.5** The IC_50_ and 1/IC_50_ values for Cu^2+^ chelating ability

|  | PERC | EARC | AERC | 95ERC | WRC | Sodium citrate |
| --- | --- | --- | --- | --- | --- | --- |
| IC_50_ (μg/mL) | 191.86±1.84 ^b^ | 319.20±6.36 ^c^ | 872.71±30.63 ^e^ | 553.34±12.59 ^d^ | 2289.11±100.00 ^f^ | 107.86±0.31^a^ |
| 1/IC_50_ (mL/μg) | 0.00521 | 0.00313 | 0.00115 | 0.00180 | 0.000437 |  |

IC_50_ value is defined as the concentration of 50% protection percentage. It was calculated by linear regression analysis and expressed as Mean±SD (n=3). The linear regression was analyzed by Origin 6.0 professional software. Means values with different superscripts in the same row are significantly different (*p<*0.05); Means values with same superscripts in the same row are not significantly different (*p<*0.05). PERC: petroleum ether extract from rhizoma *Cimicifugae*; EARC:ethyl acetate extract from rhizoma Cimicifugae; AERC: absolute ethanol extract from rhizoma *Cimicifugae*; 95ERC: 95% ethanol extract from rhizoma *Cimicifugae*; WRC: water extract from rhizoma *Cimicifugae*.

**Figure A2.6** The dose response curves of DPPH• scavenging activity. Each value is expressed as mean ± standard deviation, n =3. PERC: petroleum ether extract from rhizoma *Cimicifugae*; EARC:ethyl acetate extract from rhizoma Cimicifugae; AERC: absolute ethanol extract from rhizoma *Cimicifugae*; 95ERC: 95% ethanol extract from rhizoma *Cimicifugae*; WRC: water extract from rhizoma *Cimicifugae*.

**Table A2.6** The IC_50_ and 1/IC_50_ values for DPPH• scavenging activity

|  | PERC | EARC | AERC | 95ERC | WRC | Trolox |
| --- | --- | --- | --- | --- | --- | --- |
| IC_50_ (μg/mL) | 813.00±225.17^d^ | 227.09±10.14 ^b^ | 260.34±11.37^c^ | 254.73±4.33^c^ | 229.17±5.81^b^ | 5.13±1.19 ^a^ |
| 1/IC_50_ (mL/μg) | 0.00123 | 0.00440 | 0.00384 | 0.00393 | 0.00436 |  |

IC_50_ value is defined as the concentration of 50% protection percentage. It was calculated by linear regression analysis and expressed as Mean±SD (n=3). The linear regression was analyzed by Origin 6.0 professional software. Means values with different superscripts in the same row are significantly different (*p<*0.05); Means values with same superscripts in the same row are not significantly different (*p<*0.05). PERC: petroleum ether extract from rhizoma *Cimicifugae*; EARC:ethyl acetate extract from rhizoma Cimicifugae; AERC: absolute ethanol extract from rhizoma *Cimicifugae*; 95ERC: 95% ethanol extract from rhizoma *Cimicifugae*; WRC: water extract from rhizoma *Cimicifugae*.

**Figure A2.7** The dose response curves of ABTS^+^• scavenging activity. Each value is expressed as mean ± standard deviation, n =3. PERC: petroleum ether extract from rhizoma *Cimicifugae*; EARC:ethyl acetate extract from rhizoma Cimicifugae; AERC: absolute ethanol extract from rhizoma *Cimicifugae*; 95ERC: 95% ethanol extract from rhizoma *Cimicifugae*; WRC: water extract from rhizoma *Cimicifugae*.

**Table A2.7** The IC_50_ and 1/IC_50_ values for ABTS^+^• scavenging activity

|  | PERC | EARC | AERC | 95ERC | WRC | GSH |
| --- | --- | --- | --- | --- | --- | --- |
| IC_50_ (μg/mL) | 395.65±77.34 ^e^ | 72.10±1.32 ^b^ | 96.39±1.12 ^d^ | 94.38±2.45 ^d^ | 83.58±0.81 ^b^ | 4.76±0.28 ^a^ |
| 1/IC_50_ (mL/μg) | 0.00253 | 0.0139 | 0.0104 | 0.0106 | 0.0120 |  |

IC_50_ value is defined as the concentration of 50% protection percentage. It was calculated by linear regression analysis and expressed as Mean±SD (n=3). The linear regression was analyzed by Origin 6.0 professional software. Means values with different superscripts in the same row are significantly different (*p<*0.05); Means values with same superscripts in the same row are not significantly different (*p<*0.05). PERC: petroleum ether extract from rhizoma *Cimicifugae*; EARC:ethyl acetate extract from rhizoma Cimicifugae; AERC: absolute ethanol extract from rhizoma *Cimicifugae*; 95ERC: 95% ethanol extract from rhizoma *Cimicifugae*; WRC: water extract from rhizoma *Cimicifugae*.

**Figure A2.8** The dose response curves of Fe^3+^ reducing power assay. Each value is expressed as mean ± standard deviation, n =3. PERC: petroleum ether extract from rhizoma *Cimicifugae*; EARC:ethyl acetate extract from rhizoma Cimicifugae; AERC: absolute ethanol extract from rhizoma *Cimicifugae*; 95ERC: 95% ethanol extract from rhizoma *Cimicifugae*; WRC: water extract from rhizoma *Cimicifugae*.

**Table A2.8** The IC_50_ and 1/IC_50_ values for Fe^3+^ reducing power

|  | PERC | EARC | AERC | 95ERC | WRC | GSH |
| --- | --- | --- | --- | --- | --- | --- |
| IC_50_ (μg/mL) | 533.12±54.25 ^e^ | 159.35±1.37 ^b^ | 317.06±3.47 ^c^ | 156.19±2.12 ^b^ | 488.75±18.60 ^d^ | 51.89±1.29 ^a^ |
| 1/IC_50_ (mL/μg) | 0.00188 | 0.00628 | 0.00315 | 0.00640 | 0.00205 |  |

IC_50_ value is defined as the concentration of 50% protection percentage. It was calculated by linear regression analysis and expressed as Mean±SD (n=3). The linear regression was analyzed by Origin 6.0 professional software. Means values with different superscripts in the same row are significantly different (*p<*0.05); Means values with same superscripts in the same row are not significantly different (*p<*0.05). PERC: petroleum ether extract from rhizoma *Cimicifugae*; EARC:ethyl acetate extract from rhizoma Cimicifugae; AERC: absolute ethanol extract from rhizoma *Cimicifugae*; 95ERC: 95% ethanol extract from rhizoma *Cimicifugae*; WRC: water extract from rhizoma *Cimicifugae*.
