# Supplementary material for: Antioxidant activity and mechanism of Rhizoma Cimicifugae
Source: Chem Cent J. 2012 Nov 23;6:140. doi: 10.1186/1752-153X-6-140 (PMC3557226; doi:10.1186/1752-153X-6-140)
Supplement: Additional file 3 — Provides the calculations of contents of total phenolics, total saponins, and total sugars. [file 1752-153X-6-140-S3.doc]

Additional file 3- Calculations of chemical contents

**Antioxidant Activity and Mechanism of Rhizoma *Cimicufugae***

Xican Li*^,‡,1^, Jing Lin^‡,1^, Yaoxiang Gao^1^, Weijuan Han^1^, and Dongfeng Chen*^,2^

^1^*School of Chinese Herbal Medicine,* ^2^*School of Basic medicine, Guangzhou University of Chinese Medicine, Guangzhou, 510006, China*

^*^Corresponding author: [lixican@126.com](mailto:lixican@126.com); cdf27212@21cn.com

^‡^ Both authors contributed equally to this work.

----------------------------------------------------------------------------------------------------------------------

**Abstract**

Additional data file 3 provides detailed data concerning chemical contents of total total phenolics, total saponins, and total sugars.

The data **underlined** are cited by the main text.

**Figure A3.1** The standard curve of pyrogallol

**Table A3.1** The total phenolics contents of five extracts from rhizoma *Cimicifugae*

|  | PERC | EARC | AERC | 95ERC | WRC |
| --- | --- | --- | --- | --- | --- |
| A_760nm_ | 0.258 | 1.016 | 0.299 | 0.545 | 0.265 |
|  | 0.269 | 1.015 | 0.304 | 0.510 | 0.253 |
|  | 0.258 | 1.024 | 0.294 | 0.515 | 0.236 |
| Total phenolics | 12.92 | 64.85 | 15.73 | 32.58 | 13.40 |
|  | 13.67 | 64.78 | 16.07 | 30.18 | 12.57 |
|  | 12.92 | 65.40 | 15.38 | 30.53 | 11.41 |
| Mean±SD | 13.17±0.43^a^ | 65.01±0.34 ^d^ | 15.73±0.34 ^b^ | 31.10±1.30 ^c^ | 12.46±1.00 ^a^ |

All tests and analyses were run in triplicate. The contents of total phenolics were calculated based on the regression equation: y= 0.06945 + 36.4843 * x. y = A_760nm_ . X is the content of total phenolics. Means values with different superscripts in the same row are significantly different (*p<*0.05); Means values with same superscripts in the same row are not significantly different (*p<*0.05). PERC: petroleum ether extract from rhizoma *Cimicifugae*; EARC:ethyl acetate extract from rhizoma Cimicifugae; AERC: absolute ethanol extract from rhizoma *Cimicifugae*; 95ERC: 95% ethanol extract from rhizoma *Cimicifugae*; WRC: water extract from rhizoma *Cimicifugae*.

Figure A3.2 The standard curve of oleanolic acid

Table A3.2 The total saponins contents of five extracts from rhizoma *Cimicifugae*

|  | PERC | EARC | AERC | 95ERC | WRC |
| --- | --- | --- | --- | --- | --- |
| A_540nm_ | 0.784 | 0.302 | 0.15 | 0.15 | 0.165 |
|  | 0.652 | 0.311 | 0.144 | 0.147 | 0.162 |
|  | 0.752 | 0.287 | 0.149 | 0.149 | 0.147 |
| Total saponins | 987.24 | 382.02 | 188.14 | 188.14 | 207.27 |
|  | 818.88 | 393.49 | 180.48 | 184.31 | 203.44 |
|  | 946.43 | 362.88 | 186.86 | 186.86 | 184.31 |
| Mean±SD | 917.52±87.83^e^ | 379.46±15.46 ^d^ | 185.16±4.10 ^b^ | 180.44±1.95 ^a^ | 198.34±12.30 ^c^ |

All tests and analyses were run in triplicate. The contents of total saponins were calculated based on the regression equation: y= 0.00252 + 0.00981 * x. y = A_540nm_ . X is the content of total saponins. Means values with different superscripts in the same row are significantly different (*p<*0.05); Means values with same superscripts in the same row are not significantly different (*p<*0.05). PERC: petroleum ether extract from rhizoma *Cimicifugae*; EARC:ethyl acetate extract from rhizoma Cimicifugae; AERC: absolute ethanol extract from rhizoma *Cimicifugae*; 95ERC: 95% ethanol extract from rhizoma *Cimicifugae*; WRC: water extract from rhizoma *Cimicifugae*.

Figure A3.3 The standard curve of glucose

Table A3.3 The total sugars contents of five extracts from rhizoma *Cimicifugae*

|  | PERC | EARC | AERC | 95ERC | WRC |
| --- | --- | --- | --- | --- | --- |
| A_490nm_ | 0.297 | 0.210 | 0.638 | 0.59 | 0.769 |
|  | 0.245 | 0.210 | 0.619 | 0.591 | 0.756 |
|  | 0.227 | 0.175 | 0.605 | 0.621 | 0.844 |
| Total sugars | 280.43 | 187.87 | 643.19 | 592.13 | 782.55 |
|  | 225.11 | 187.87 | 622.98 | 593.19 | 768.72 |
|  | 205.96 | 150.64 | 608.09 | 625.11 | 862.34 |
| Mean±SD | 237.16±38.67^b^ | 175.46±21.50 ^a^ | 624.75±17.62 ^c^ | 603.48±18.74 ^c^ | 804.54±50.53 ^d^ |

All tests and analyses were run in triplicate. The contents of total sugars were calculated based on the regression equation: y= 0.0334 + 0.02352 * x. y = A_540nm_ . X is the content of total sugars. Means values with different superscripts in the same row are significantly different (*p<*0.05); Means values with same superscripts in the same row are not significantly different (*p<*0.05). PERC: petroleum ether extract from rhizoma *Cimicifugae*; EARC:ethyl acetate extract from rhizoma Cimicifugae; AERC: absolute ethanol extract from rhizoma *Cimicifugae*; 95ERC: 95% ethanol extract from rhizoma *Cimicifugae*; WRC: water extract from rhizoma *Cimicifugae*.
