# Supplementary material for: Antioxidant activity and mechanism of Rhizoma Cimicifugae
Source: Chem Cent J. 2012 Nov 23;6:140. doi: 10.1186/1752-153X-6-140 (PMC3557226; doi:10.1186/1752-153X-6-140)
Supplement: Additional file 4 — Shows HPLC figures and peak areas. [file 1752-153X-6-140-S4.doc]

Additional file 4-HPLC figures and peak areas

**Antioxidant Activity and Mechanism of Rhizoma *Cimicifugae***

Xican Li*^,‡,1^, Jing Lin^‡,1^, Yaoxiang Gao^1^, Weijuan Han^1^, and Dongfeng Chen*^,2^

^1^*School of Chinese Herbal Medicine,* ^2^*School of Basic medicine, Guangzhou University of Chinese Medicine, Guangzhou, 510006, China*

^*^Corresponding author: lixican@126.com

^‡^ Both authors contributed equally to this work.

----------------------------------------------------------------------------------------------------------------------

**Abstract**

The Additional file 4 shows HPLC figures.


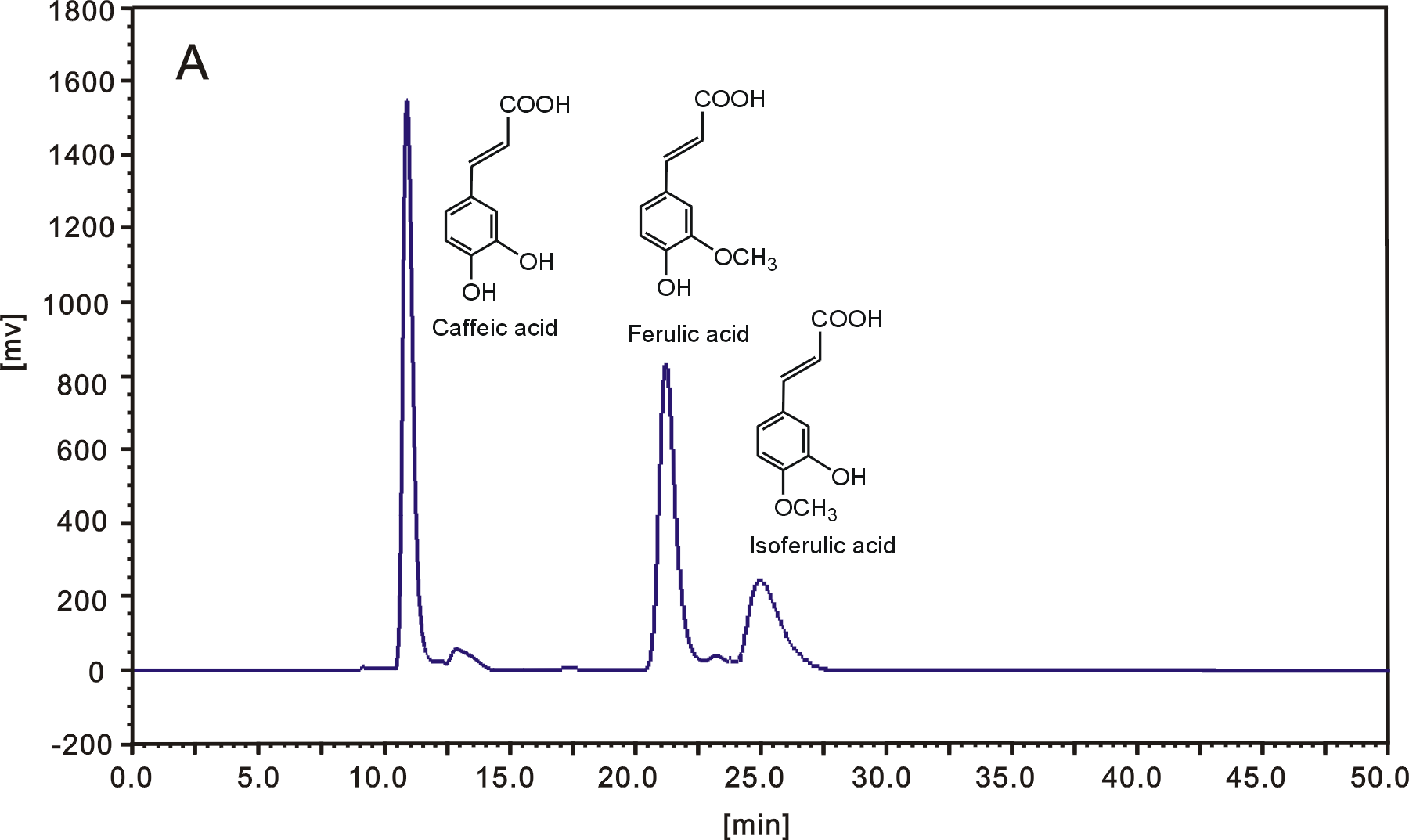


Figure A4.1 The HPLC profile of standard caffeic acid, ferulic acid, and isoferulic acid

HPLC analysis was performed on a Syltech P510 system (Los Angeles, California, USA) equipped with Dikma Diamonsil C_18_ (250 mm×4.6 mm, 5 μm) (Beijing, China). The mobile phase consisted of acetonitrile-0.5 % acetic acid in water (17:83, v: v), the flow rate was 1.0 mL/min, injection volume was 30 µL and absorption was measured at 316 nm.


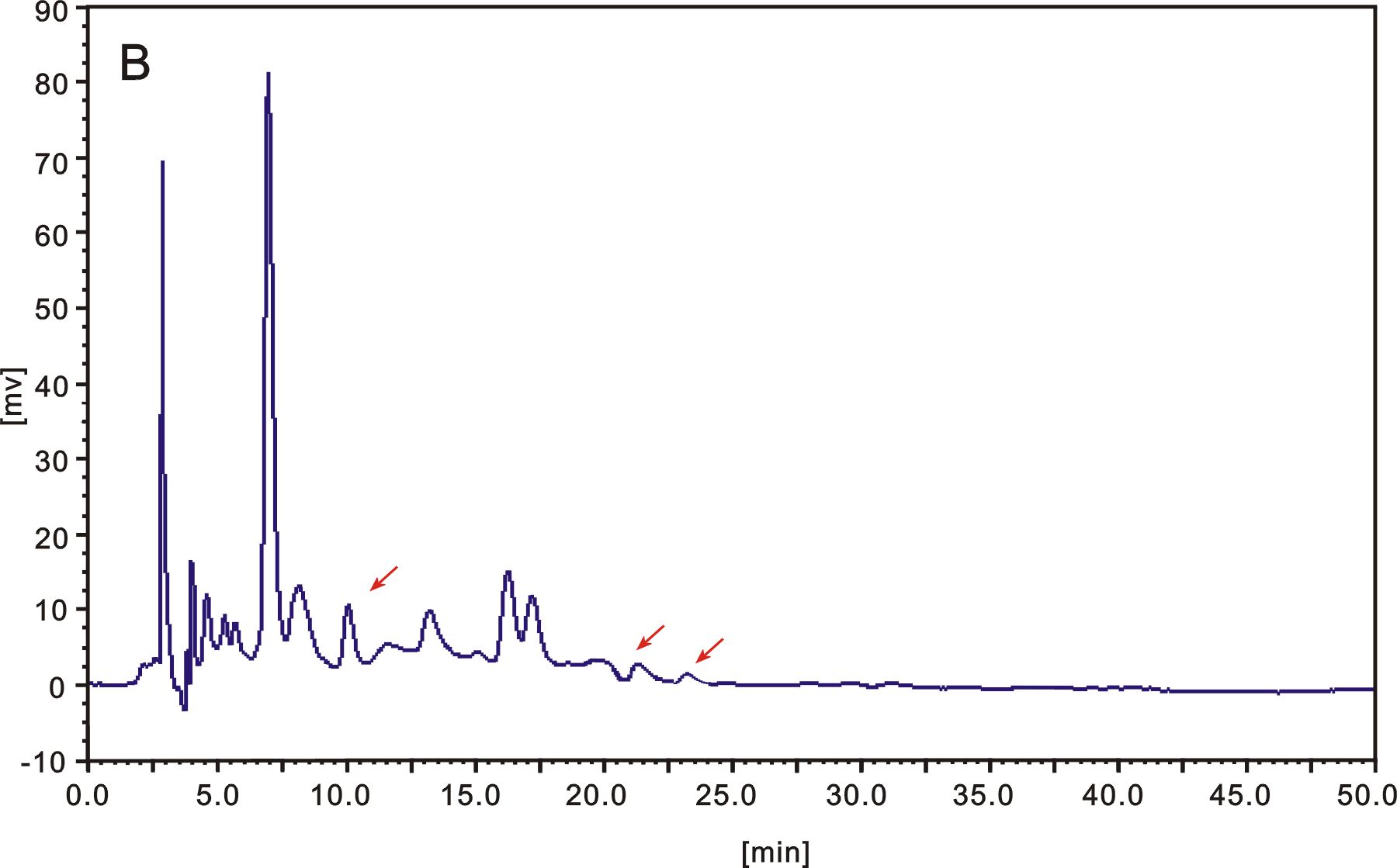


Figure A4.2 The HPLC profile of PERC (petroleum ether extract of rhizoma *Cimicifugae*)

HPLC analysis was performed on a Syltech P510 system (Los Angeles, California, USA) equipped with Dikma Diamonsil C_18_ (250 mm×4.6 mm, 5 μm) (Beijing, China). The mobile phase consisted of acetonitrile-0.5 % acetic acid in water (17:83, v: v), the flow rate was 1.0 mL/min, injection volume was 30 µL and absorption was measured at 316 nm.


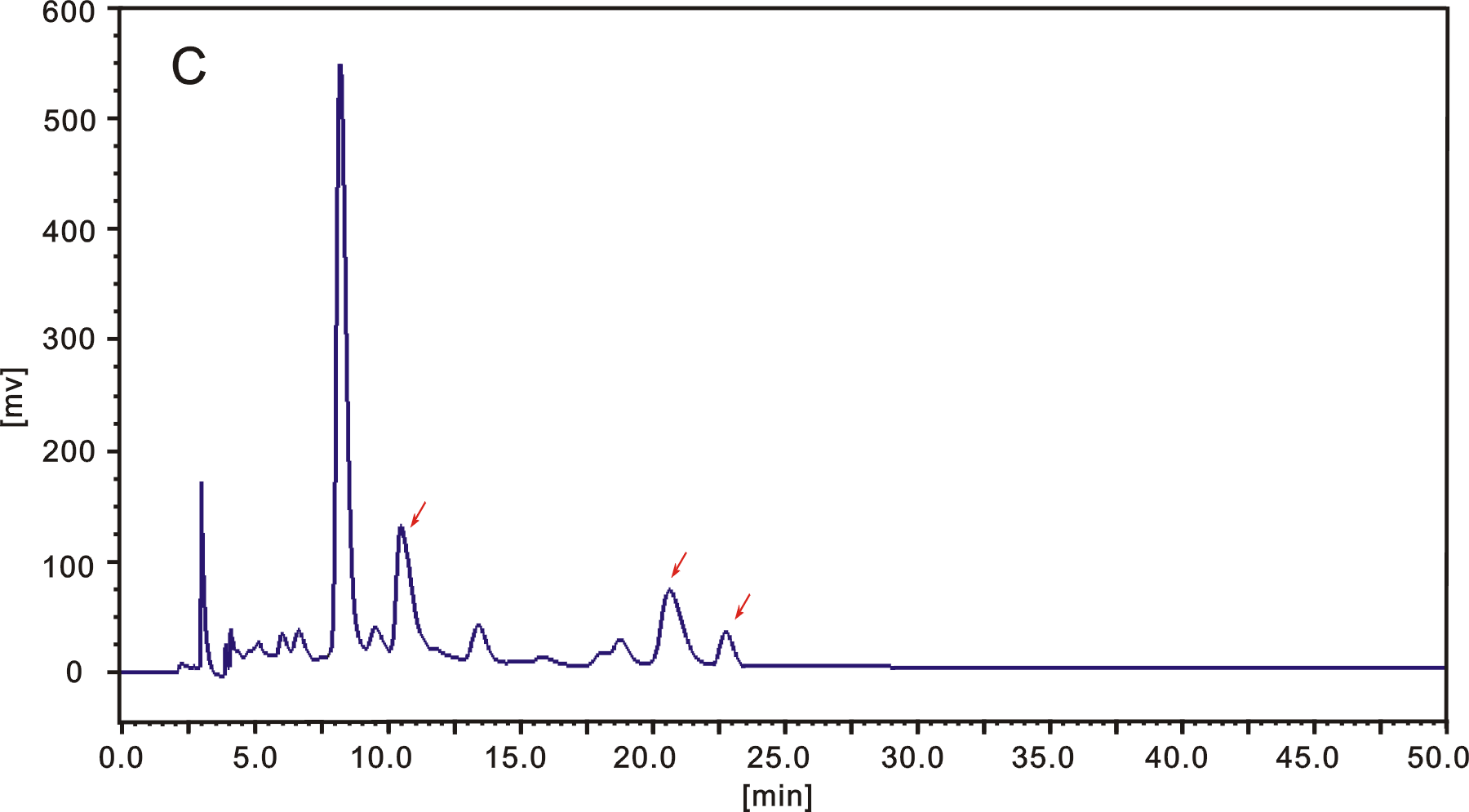


Figure A4.3 The HPLC profile of EARC (ethyl acetate extract of rhizoma *Cimicifugae*)

HPLC analysis was performed on a Syltech P510 system (Los Angeles, California, USA) equipped with Dikma Diamonsil C_18_ (250 mm×4.6 mm, 5 μm) (Beijing, China). The mobile phase consisted of acetonitrile-0.5 % acetic acid in water (17:83, v: v), the flow rate was 1.0 mL/min, injection volume was 30 µL and absorption was measured at 316 nm.


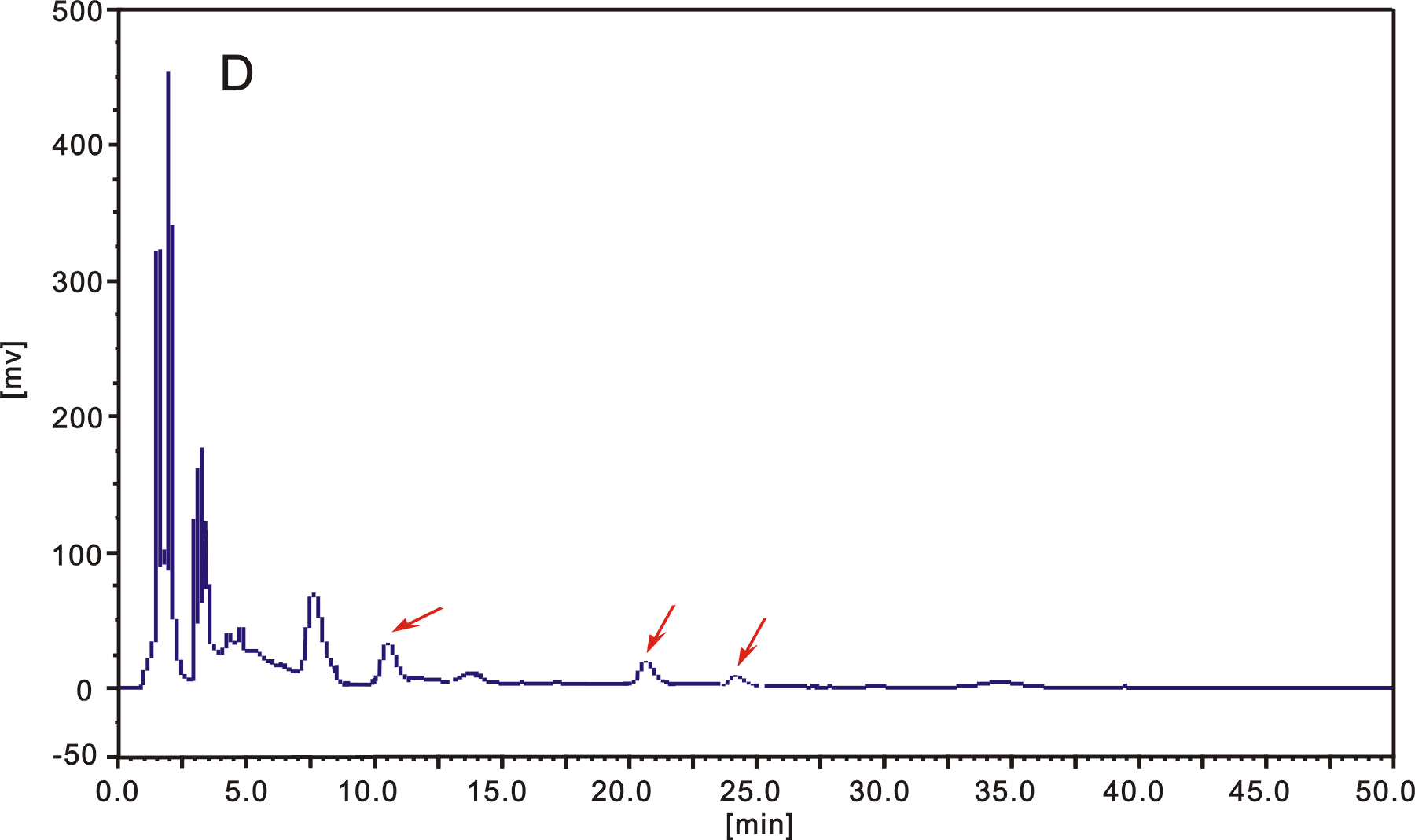


**Figure A4.4** The HPLC profile of AERC (absolute ethanol extract of rhizoma *Cimicifugae)*

HPLC analysis was performed on a Syltech P510 system (Los Angeles, California, USA) equipped with Dikma Diamonsil C_18_ (250 mm×4.6 mm, 5 μm) (Beijing, China). The mobile phase consisted of acetonitrile-0.5 % acetic acid in water (17:83, v: v), the flow rate was 1.0 mL/min, injection volume was 30 µL and absorption was measured at 316 nm.


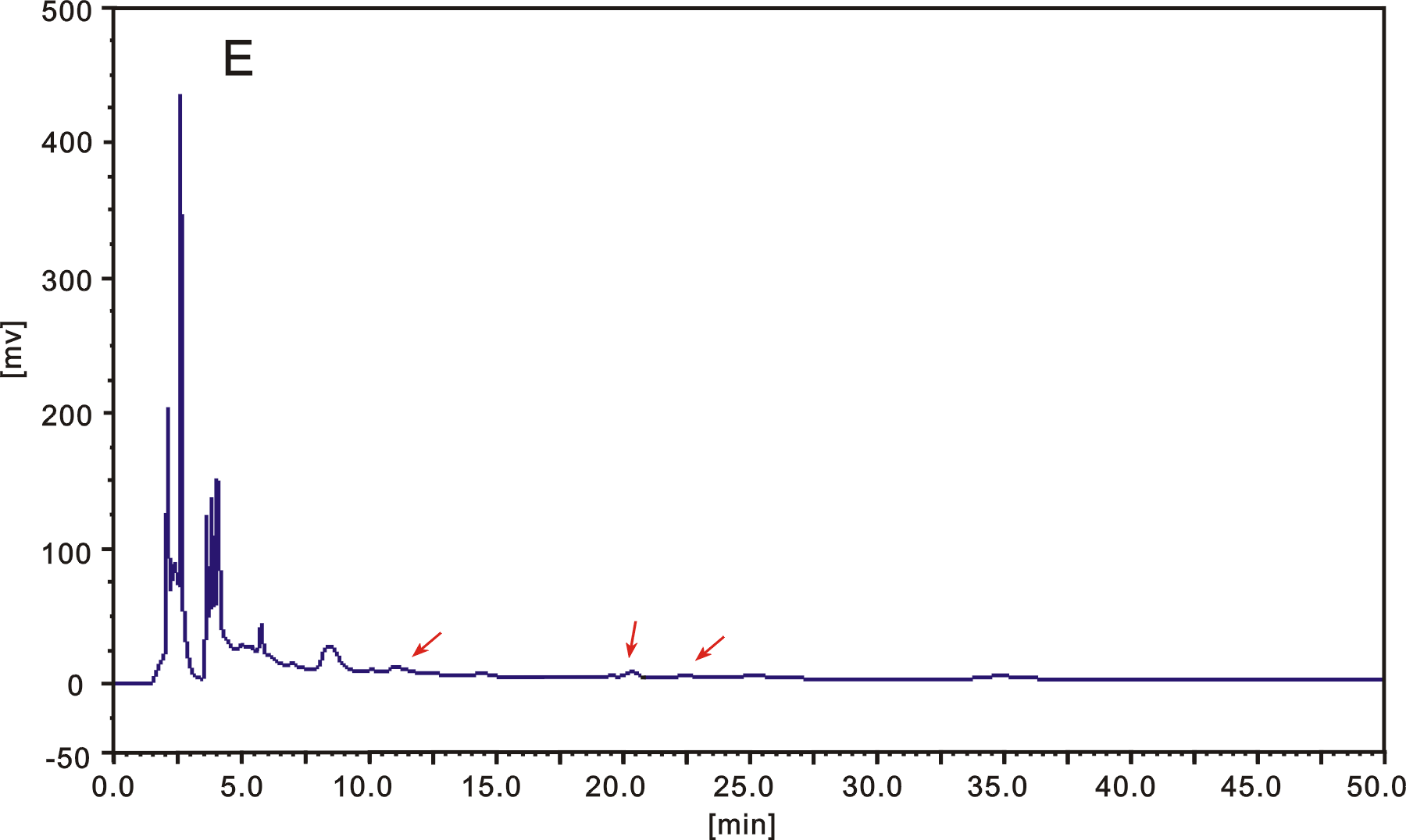


**Figure A4.5** The HPLC profile of 95ERC (95% ethanol extract of rhizoma *Cimicifugae)*

HPLC analysis was performed on a Syltech P510 system (Los Angeles, California, USA) equipped with Dikma Diamonsil C_18_ (250 mm×4.6 mm, 5 μm) (Beijing, China). The mobile phase consisted of acetonitrile-0.5 % acetic acid in water (17:83, v: v), the flow rate was 1.0 mL/min, injection volume was 30 µL and absorption was measured at 316 nm.


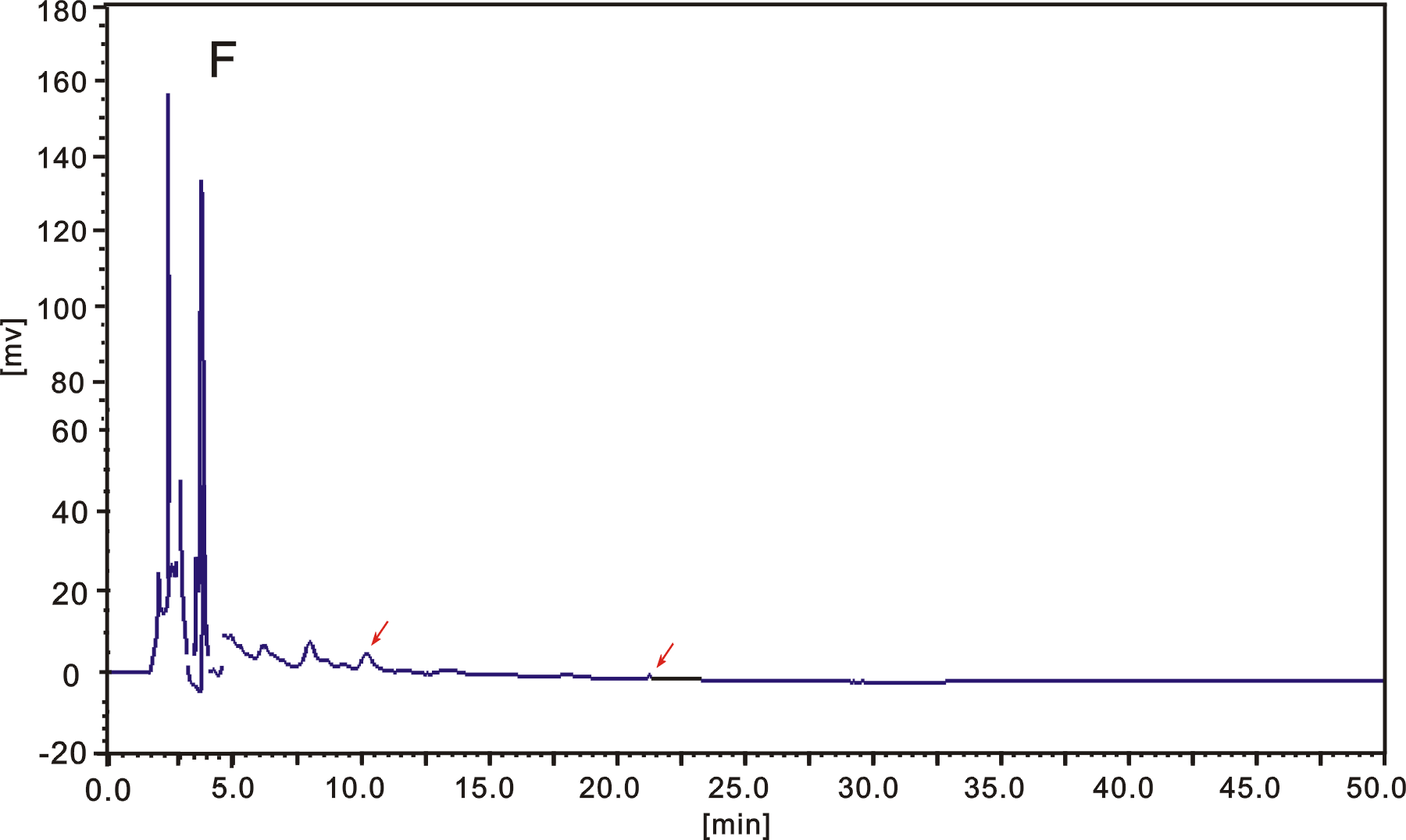


**Figure A4.6** The HPLC profile of WRC (water extract of rhizoma *Cimicifugae)*

HPLC analysis was performed on a Syltech P510 system (Los Angeles, California, USA) equipped with Dikma Diamonsil C_18_ (250 mm×4.6 mm, 5 μm) (Beijing, China). The mobile phase consisted of acetonitrile-0.5 % acetic acid in water (17:83, v: v), the flow rate was 1.0 mL/min, injection volume was 30 µL and absorption was measured at 316 nm.
