# Supplementary material for: Antioxidant activity and mechanism of Rhizoma Cimicifugae
Source: Chem Cent J. 2012 Nov 23;6:140. doi: 10.1186/1752-153X-6-140 (PMC3557226; doi:10.1186/1752-153X-6-140)
Supplement: Additional file 5 — Includes all correlation graphs. [file 1752-153X-6-140-S5.doc]

Additional file 5-All correlation graphs

**Antioxidant Activity and Mechanism of Rhizoma *Cimicufugae***

Xican Li*^,‡,1^, Jing Lin^‡,1^, Yaoxiang Gao^1^, Weijuan Han^1^, and Dongfeng Chen*^,2^

^1^*School of Chinese Herbal Medicine,* ^2^*School of Basic Medical Science, Guangzhou University of Chinese Medicine, Guangzhou, 510006, China*

^*^Corresponding author: lixican@126.com;CDF27212@21CN.COM

^‡^ Both authors contributed equally to this work.

----------------------------------------------------------------------------------------------------------------------

**Abstract**

The Additional file 5 includes all correlation graphs (48 in total).

The data **underlined** are cited by the main text.

Figure A5.1 The correlation graphs between total phenolics and antioxidant levels

(A) for LPO assay, (B) for DNA assay, (C) for ·OH assay, (D) for·O_2_^-^ assay, (E) for chelating ability assay, (F) for DPPH assay, (G) for ABTS assay, (H) for reducing power assay

Figure A5.2 The correlation graphs between caffeic acid and antioxidant levels

(A) for LPO assay, (B) for DNA assay, (C) for ·OH assay, (D) for·O_2_^-^ assay, (E) for chelating ability assay, (F) for DPPH assay, (G) for ABTS assay, (H) for reducing power assay

Figure A5.3 The correlation graphs between ferulic acid and antioxidant levels

(A) for LPO assay, (B) for DNA assay, (C) for ·OH assay, (D) for·O_2_^-^ assay, (E) for chelating ability assay, (F) for DPPH assay, (G) for ABTS assay, (H) for reducing power assay

Figure A5.4 The correlation graphs between isoferulic acid and antioxidant levels

(A) for LPO assay, (B) for DNA assay, (C) for ·OH assay, (D) for·O_2_^-^ assay, (E) for chelating ability assay, (F) for DPPH assay, (G) for ABTS assay, (H) for reducing power assay

Figure A5.5 The correlation graphs between total sugars and antioxidant levels

(A) for LPO assay, (B) for DNA assay, (C) for ·OH assay, (D) for·O_2_^-^ assay, (E) for chelating ability assay, (F) for DPPH assay, (G) for ABTS assay, (H) for reducing power assay

Figure A5.6 The correlation graphs between total saponins and antioxidant levels

(A) for LPO assay, (B) for DNA assay, (C) for ·OH assay, (D) for·O_2_^-^ assay, (E) for chelating ability assay, (F) for DPPH assay, (G) for ABTS assay, (H) for reducing power assay
